# Comparative Analysis of Glycemic and Lipid Profiles in Newly Diagnosed Males and Females With Type 2 Diabetes Mellitus

**DOI:** 10.7759/cureus.50101

**Published:** 2023-12-07

**Authors:** Haider A Alidrisi, Ali A Al-Ibadi, Jaafer S Al-Saidi, Mohammed A Alsawad, Ahmed A Jameel, Ahmed W Al-Shati

**Affiliations:** 1 Diabetes and Endocrinology, College of Medicine, Faiha Specialized Diabetes, Endocrine, and Metabolism Center, University of Basrah, Basrah, IRQ; 2 College of Medicine, Faiha Specialized Diabetes, Endocrine, and Metabolism Center, University of Basrah, Basrah, IRQ

**Keywords:** total cholesterol level, triglyceride, high density lipoprotein-cholesterol, low density lipoprotein-cholesterol, random blood glucose (rbg), fasting blood glucose (fbg), glycosylated hemoglobin (hba1c), types 2 diabetes

## Abstract

Background

Age- and gender-based differences in diabetes demographic characteristics have been studied in many types of research. These differences extend further to diabetes-related comorbidities. Dyslipidemia is a common complication associated with diabetes and causes a substantial increase in cardiovascular morbidity. The study aims to compare the pattern of dyslipidemia between males and females among different age categories in newly diagnosed type 2 diabetes mellitus (T2DM).

Methodology

A retrospective database study was conducted at Faiha Specialized Diabetes, Endocrine, and Metabolism Center (FDEMC), Basrah, Southern Iraq. We included adult patients with newly diagnosed and drug naïve T2DM between January 2018 and October 2022. Patients’ data in the form of body mass index (BMI), hemoglobin A1c (HbA1c), fasting blood glucose (FBG), random blood glucose (RBG), total cholesterol (TC), triglyceride (TG), high-density lipoprotein cholesterol (HDL-C), and low-density lipoprotein cholesterol (LDL-C) were used for comparisons.

Results

Below the age of 35, males exhibited significantly higher levels of HbA1c, FBG, and TG compared to females, along with a significantly lower level of HDL-C. However, there were no significant differences in BMI, RBG, TC, and LDL-C. Between the ages of 35 and 44, females in this study demonstrated significantly higher BMI and HDL-C levels, while males exhibited higher levels of HbA1c, FBG, RBG, and TG. However, there were no significant differences observed in TC and LDL-C levels. Similar results were found among the age group 45 to 55, with the only exception being FBG, which became nonsignificant. In patients between 55 and 64 years old, BMI, HDL-C, and TC were significantly higher in females (*P *< 0.05). In patients aged above 65 years, BMI and HDL-C remained significantly higher in females, while RBG was significantly higher in males. No significant differences were observed among other parameters (HbA1c, TG, TC, and LDL-C).

Conclusions

In patients aged 54 years and younger, males were significantly more likely to have severe hyperglycemia, higher TG, and lower HDL-C compared to females at the time of T2DM diagnosis. In older patients, this pattern is lost, with only a significantly lower HDL-C observed.

## Introduction

Diabetes mellitus is a chronic metabolic disorder characterized by high blood sugar levels. It is caused by a combination of insulin resistance, which occurs when the body's cells do not effectively utilize insulin due to many underlying causes, and insufficient insulin production by the pancreas that results from progressive loss of pancreatic islets activity or number [1]. In recent years, diabetes mellitus has emerged as a serious global concern. As of 2021, an estimated 537 million adults aged 20-79 years were living with diabetes, and this number is projected to rise to 643 million by 2030. This increase in the total number of individuals with the disease is expected to exacerbate the burden of diabetes-related deaths, currently totaling 6.7 million, as well as long-term morbidities and disabilities. All of these factors will continue to impose significant strain on healthcare systems [2]. For the prevalence in our area of the Middle East and North Africa, the burden is that of 73 million affected individuals with a projected estimate of 95 million by 2030. The prevalence in Iraq specifically was 4.6 million (10.7%) of the population, and the estimate is projected to increase to 5.8 million (11.6%) by 2030 [2]. The prevalence is also influenced by factors such as old age, specific ethnic groups, and a higher occurrence in men when compared to women [3]. The development of type 2 diabetes mellitus (T2DM) may be influenced by several factors, including increasing age, being overweight, females with a history of gestational diabetes, polycystic ovary syndrome, leading a sedentary lifestyle, and having a family history of diabetes (genetic factors) [1,2].

Serious and long-term complications are highly prevalent in diabetic patients as they are at an increased risk of developing such complications which include CVDs, nephropathy, neuropathy, retinopathy, and even loss of vision [1]. Dyslipidemia is a common complication associated with diabetes. Lipid indices dysregulation causes a substantial increase in cardiovascular complications in patients with T2DM, and this comes from the fact that dysregulation of lipid indices plays a role in the development and progression of atherosclerosis [1,4]. The dysregulation of lipids involves both qualitative and quantitative kinetics. Qualitative abnormalities result from increased production of small dense low-density lipoprotein cholesterol (LDL-C), very large VLDL-C subfractions, and an increase in the TG content of those particles. Quantitatively, an increase in triglyceride (TG) contributes to a decrease in clearance and catabolism. Additionally, there is a decrease in high-density lipoprotein cholesterol (HDL-C) particles [1,4]. This alteration in lipid parameters is associated with an increased risk of both macro- and microvascular complications in individuals with diabetes [1,5]. To minimize or, at the very least, diagnose complications linked to poor glycemic control as early as possible, it is essential to maintain frequent follow-ups for these patients. Multiple tools are available for monitoring, including the use of glucometer devices by the patients themselves, fasting blood glucose (FBG) measurements in clinics, and continuous glucose monitoring (CGM) systems [1]. However, these tools do not provide an idea about the glycemic control over a long period, which is required in cases of noncompliance and failure of treatment; thus, the most commonly used tool for such monitoring is hemoglobin A1c (HbA1c) [6], which provides an idea about the glycemic control for the prior three months and this comes handy in the assessment of treatment regime in the long run [7,8].

The differences in lipid indices between males and females can be a major cause of the variation in long-term complications of diabetes, especially cardiovascular diseases (CVD). Generally, females have higher levels of HDL-C and lower TG, as well as total cholesterol (TC) to HDL ratio, when compared to males [5,8]. These differences may be attributed to the different levels of circulating sex hormones, namely, estrogens and progesterone. One of the effects is the increase in the production of apolipoprotein A-I (apo A-I), which explains the higher levels of HDL-C in females [8]. However, in females, the risk of CVD tends to increase with age, particularly above 50, when compared to males. This is attributed to a rise in LDL production by around 15% to 25%, which stems from the decline in circulating estrogen levels. Also, lipoprotein A is associated with an increased risk of atherogenesis and thrombogenesis, consequently elevating the overall risk of CVDs and strokes, although no significant association was found between lipoprotein A and an increase in age in females [8]. The differences in lipid indices between genders and age groups among the studied cases with T2DM were notably elevated. The associated risk was three times higher compared to non-diabetics within the same age group. The heightened risk further emphasizes the importance of the study in exploring the significance of lipid index abnormalities among patients with T2DM across different age groups and genders [5,8]. Building on the results obtained from this study and previous studies, we can further refine and enhance the assessment of both macro and microvascular complications, particularly those involving the cardiovascular system, in patients with T2DM. In improving the assessment and recognition of complications, a more effective management regime can be reached for different genders and age groups, depending on the risk posed by each variable [8].

## Materials and methods

This retrospective study was conducted from June to September 2022 at the Faiha Specialized Diabetes Endocrine and Metabolism Center (FDEMC) in Basrah, Southern Iraq. Database retrieval involved patients’ records from January 2018 to October 2022. The inclusion criteria encompassed adult patients aged 18 years or older who were newly diagnosed with T2DM and were not on any glucose-lowering drugs. We excluded patients with pregnancy, other endocrine diseases (thyroid, pituitary, and adrenal based on clinical and hormonal evaluations that were done through time in the center), familial hypertriglyceridemia, familial hypercholesteremia, and patients on lipid-lowering or oral contraceptive drugs. Ethical approval was obtained from the FDEMC Research Committee (ref #23/12/22).

We included a total of 1,954 patients, comprising 1,021 (52%) males and 933 (48%) females. The patients were categorized into five age groups: less than 34 years old, 35-44 years old, 45-54 years old, 55-64 years old, and 65 years old and above. Patients’ baseline data that were compared between males and females were body mass index (BMI), hemoglobin A1c (HbA1c), FBG, random blood glucose (RBG), TGs, HDL-C, TC, and LDL-C cholesterol.

To determine the significance of differences in the study variables between males and females within each age group, the independent Student's t-test was utilized for equally distributed variables, which were HbA1c, TG, HDL-C, and TC. This method was applied to variables that satisfied the assumption of equal variances according to Levene's test. In cases where the assumption of equal variances was not met, the t-test was conducted using Welch's t-test, which does not assume equal variances. Additionally, a nonparametric test, the Mann-Whitney U-test, was used to determine the significance of the differences between males and females in the not equally distributed variables, which were body mass index (BMI), FBG, RBG, and LDL-C. The values in this study were presented as mean (M) and standard deviation (SD). A *P*-value less than 0.05 was considered statistically significant.

## Results

Age group: <35 years

Table 1 summarizes the results of the comparison of the variables between males and females aged less than 35 years. There were 218 patients, with 114 (52%) males and 104 (48%) females in the age group. Males had significantly higher HbA1c, FBG, and TG and significantly lower HDL-C. No significant differences were seen in BMI, RBG, TC, and LDL-C.

**Table 1:** Comparisons of the variables between males and females newly diagnosed with type 2 diabetes of less than 35 years old. BMI, body mass index; HbA1c, hemoglobin A1c; FBG, fasting blood glucose; RBG, random blood glucose; TG, triglyceride; HDL-C, high-density lipoprotein cholesterol; TC, total cholesterol; LDL-C, low-density lipoprotein cholesterol; *n*, number of patients; M, mean; SD, standard deviation

Variable	*n* (%)		M ± SD		*P*-value
	Males	Females	Males	Females	
BMI (kg/m^2^)	114 (52.29)	102 (47.22)	29.21 ± 6.0	30.50 ± 6.50	0.1
HbA1c (%)	114 (52.29)	104 (47.71)	10.24 ± 2.86	8.39 ± 2.85	0.000003
FBG (mg/dL)	78 (46.15)	91 (53.85)	237.59 ± 103.5	166.99 ± 75.34	0.000003
RBG (mg/dL)	36 (70.59)	15 (29.41)	269.83 ± 119.3	241.47 ± 141.11	0.4
TG (mg/dL)	114 (52.29)	104 (47.71)	180.68 ± 86.13	147.69 ± 72.31	0.003
HDL-C (mg/dL)	114 (52.29)	104 (47.71)	37.55 ± 7.93	44.48 ± 9.12	<0.0001
TC (mg/dL)	114 (52.29)	104 (47.71)	172.06 ± 37.56	175.25 ± 40.69	0.5
LDL-C (mg/dL)	114 (52.29)	104 (47.71)	118.07 ± 33.49	119.06 ± 39.84	0.8

Age group: 35-44 years

Table 2 summarizes the results of the comparison of variables between males and females aged 35-44 years. There were 515 patients, with 264 (52%) males and 251 (48%) females. Males exhibited significantly higher levels of HbA1c, FBG, RBG, and TG, and significantly lower BMI and HDL-C. However, no significant differences were observed in TC and LDL-C.

**Table 2:** Comparisons of variables between males and females newly diagnosed with type 2 diabetes in the 35-44 years age group. BMI, body mass index; HbA1c, hemoglobin A1c; FBG, fasting blood glucose; RBG, random blood glucose; TG, triglyceride; HDL-C, high-density lipoprotein cholesterol; TC, total cholesterol; LDL-C, low-density lipoprotein cholesterol; *n*, number of patients; M, mean; SD, standard deviation

Variable	*n* (%)		M ± SD		*P*-value
	Males	Females	Males	Females	
BMI (kg/m^2^)	263 (51.17)	251 (48.83)	30.10 ± 5.1	33.60 ± 6.0	<0.0001
HbA1c (%)	264 (51.26)	251 (48.74)	9.52 ± 2.65	8.73 ± 2.44	0.0004
FBG (mg/dL)	207 (50)	207 (50)	206.58 ± 91.5	175.03 ± 74.0	0.0001
RBG (mg/dL)	58 (53.70)	50 (46.30)	265.34 ± 123.17	194.88 ± 82.9	0.002
TG (mg/dL)	264 (51.26)	251 (48.74)	191.78 ± 78.02	163.94 ± 74.54	0.00009
HDL-C (mg/dL)	264 (51.26)	251 (48.74)	37.97 ± 7.96	42.09 ± 9.23	<0.0001
TC (mg/dL)	264 (51.26)	251 (48.74)	184.69 ± 39.69	185.58 ± 37.07	0.7
LDL-C (mg/dL)	264 (51.26)	251 (48.74)	128.47 ± 37.0	127.33 ± 35.4	0.7

Age group: 45-54 years

Table 3 summarizes the results of the comparison of the variables between males and females of 45-54 years old. There were 664 patients, with 344 (52%) males and 320 (48%) females. Males had significantly higher HbA1c, FBG, RBG, and TG, and significantly lower BMI and HDL-C. No significant differences were seen in TC and LDL-C.

**Table 3:** Comparisons of variables between males and females newly diagnosed with type 2 diabetes in the 45-54 years age group. BMI, body mass index; HbA1c, hemoglobin A1c; FBG, fasting blood glucose; RBG, random blood glucose; TG, triglyceride; HDL-C, high-density lipoprotein cholesterol; TC, total cholesterol; LDL-C, low-density lipoprotein cholesterol; *n*, number of patients; M, mean; SD, standard deviation

Variable	*n* (%)		M ± SD		*P*-value
	Males	Females	Males	Females	
BMI (kg/m^2^)	340 (51.83)	316 (48.17)	30.48 ± 5.5	33.90 ± 5.6	<0.0001
HbA1c (%)	344 (51.81)	320 (48.19)	9.68 ± 2.65	9.14 ± 2.69	0.009
FBG (mg/dL)	264 (51.76)	246 (48.24)	202.83 ± 93.7	188.09 ± 83.9	0.06
RBG (mg/dL)	86 (53.09)	76 (46.91)	263.30 ± 123.0	204.78 ± 119.7	0.0004
TG (mg/dL)	344 (51.81)	320 (48.19)	180.74 ± 72.17	163.94 ± 67.61	0.002
HDL-C (mg/dL)	344 (51.81)	320 (48.19)	38.17 ± 7.94	43.44 ± 8.99	<0.0001
TC (mg/dL)	344 (51.81)	320 (48.19)	184.73 ± 40.06	187.15 ± 40.85	0.4
LDL-C (mg/dL)	344 (51.81)	320 (48.19)	127.41 ± 36.4	129.67 ± 38.4	0.4

Age group: 55-64 years

Table 4 summarizes the results of the comparison of the variables between males and females 55-64 years old. There were 355 participants, with 180 (51%) males and 175 (49%) females. Males had significantly lower BMI and HDL-C. No significant differences were seen in HbA1c, FBG, RBG, TG, TC, and LDL-C.

**Table 4:** Comparisons of variables between males and females newly diagnosed with type 2 diabetes in the 55-64 years age group. BMI, body mass index; HbA1c, hemoglobin A1c; FBG, fasting blood glucose; RBG, random blood glucose; TG, triglyceride; HDL-C, high-density lipoprotein cholesterol; TC, total cholesterol; LDL-C, low-density lipoprotein cholesterol; *n*, number of patients; M, mean; SD, standard deviation

Variable	*n* (%)		M ± SD		*P*-value
	Males	Females	Males	Females	
BMI (kg/m^2^)	177 (50.86)	171 (49.14)	29.85 ± 4.5	33.16 ± 6.2	<0.0001
HbA1c (%)	180 (50.70)	175 (49.30)	9.58 ± 2.52	9.28 ± 2.82	0.2
FBG (mg/dL)	134 (49.63)	136 (50.37)	198.89 ± 84.2	182.33 ± 80.4	0.1
RBG (mg/dL)	48 (54.55)	40 (45.45)	281.31 ± 155.2	240.0 ± 135.5	0.1
TG (mg/dL)	180 (50.70)	175 (49.30)	180.47 ± 72.17	179.38 ± 64.75	0.8
HDL-C (mg/dL)	180 (50.70)	175 (49.30)	39.43 ± 8.16	44.13 ± 8.81	<0.0001
TC (mg/dL)	180 (50.70)	175 (49.30)	182.29 ± 41.32	192.47 ± 44.01	0.02
LDL-C (mg/dL)	180 (50.70)	175 (49.30)	124.53 ± 38.5	133.17 ± 48.37	0.06

Age group: 65 years and older

Table 5 summarizes the results of the comparison of the variables between males and females in the 55-64 years age group. There were 202 participants, with 119 (59%) males and 83 (41%) females. Males exhibited a significantly lower BMI only, with no significant differences observed in HbA1c, FBG, RBG, TG, HDL-C, TC, and LDL-C.

**Table 5:** Comparisons of variables between males and females newly diagnosed with type 2 diabetes aged 65 years and older. BMI, body mass index; HbA1c, hemoglobin A1c; FBG, fasting blood glucose; RBG, random blood glucose; TG, triglyceride; HDL-C, high-density lipoprotein cholesterol; TC, total cholesterol; LDL-C, low-density lipoprotein cholesterol; *n*, number of patients; M, mean; SD, standard deviation

Variable	*n* (%)		M ± SD		*P*-value
	Males	Females	Males	Females	
BMI (kg/m^2^)	117 (59.39)	80 (40.61)	28.57 ± 4.7	30.66 ± 4.9	0.002
HbA1c (%)	119 (58.91)	83 (41.09)	9.81 ± 2.61	9.07 ± 2.79	0.05
FBG (mg/dL)	82 (58.99)	57 (41.01)	191.37 ± 78.4	196.82 ± 97.0	0.7
RBG (mg/dL)	37 (57.81)	27 (42.19)	284.32 ± 128.6	224.78 ± 105.9	0.05
TG (mg/dL)	119 (58.91)	83 (41.09)	176.28 ± 74.28	174.84 ± 60.02	0.8
HDL-C (mg/dL)	119 (58.91)	83 (41.09)	40.26 ± 9.43	43.45 ± 8.14	0.01
TC (mg/dL)	119 (58.91)	83 (41.09)	180.16 ± 46.05	190.66 ± 42.41	0.1
LDL-C (mg/dL)	119 (58.91)	83 (41.09)	122.43 ± 42.8	130.11 ± 41.1	0.2

## Discussion

From the data, males had higher HbA1c levels than females. There was a significant difference in HbA1c within all age groups, except for those between 55-64 and above 65 years old, who did not show a significant difference. These results were consistent with a cross-sectional study in China on 18,265 patients that showed higher HbA1c in males[9]. In another retrospective study from Iraq, female gender was one of the independent predictors of higher HbA1c [10]. However, our study included only data from the first diagnosis of T2DM. Regarding TG, males had higher levels in all age groups, with the difference being significant only in those aged less than 35, 35-44, and 45-54 years old. However, it's noteworthy that the levels were within the upper limits of normal. This is consistent with a 2018 study that found males to have significantly higher TG levels than females [11]. In contrast, a study in China indicated that females had significantly higher TG levels than males [12], while another study in India revealed that females were insignificantly higher than males [13]. TC levels were higher in females, with the only age group showing a significant difference being 55-64 years old, and the levels were within the upper normal range for all age groups. Many other studies showed similar results with females higher than males, and one of them showed a significant difference in the elderly population [11-14], while others showed no significant difference without specifying the age groups [13,15,16]. The finding that females had higher TC may be attributed to the presence of higher sex hormones, particularly E2, and its impact on lipid metabolism [15,17]. One study concluded that the TC and TG levels are significantly higher in postmenopausal women, which may be due to low levels of estrogen [18]. A study in India revealed females insignificantly higher in TG and TC and significantly higher in HDL-C and LDL-C [13]. While females were significantly higher in TC, TG, HDL-C, and LDL-C in a study in China [12], HDL-C levels were significantly higher in females than males, who showed below-normal levels of HDL-C. This trend was observed across all age groups. This is consistent with two studies showing females to have significantly higher HDL-C than males (the age group 41-50 showed higher HDL-C in males) [13,15]. This could be attributed to high estrogen levels in females and its effect on HDL-C metabolism. Oral estrogen was found to increase HDL-C by up to 5% and decrease LDL-C by 15% [19, 20]. There were no significant differences between males and females in LDL-C levels and the mean was higher in females, except for the 35-44-year-old age group where males had higher means. The same study in Ghana in 2018 found no significant difference between males and females in LDL-C levels. It revealed equal levels between postmenopausal women and men, while premenopausal women had lower LDL-C levels than men. This was attributed to the effect of estrogen, which increases hepatic LDL-C receptors and leads to the rapid clearance of LDL-C particles from the circulation in premenopausal women [15]. Another study found that females with T2DM were 42% more likely than males to have LDL-C >= 130 mg/dL, 14% for HbA1c >= 9%, and 50% for BMI >= 30 kg/m^2^ despite the use of lipid-lowering drugs [17]. While BMI was higher in females across all age groups, it was significant in all groups except for less than 35 groups. This significant difference was consistent with the results of a 2008 study based on Scottish Care Information Diabetes Collaboration (SCI-DC) data [21]. The study found that at most ages between 30 and 90 years old, women had a higher BMI at the diagnosis of T2DM. This was explained by the fact that, for a given BMI, women are more insulin-sensitive than men. However, it's noteworthy that the difference is nonsignificant under 35 years of age [21]. It's important to mention that Logue did not include diabetic patients under 30 years old in this study. Another study also found that BMI was lower in males than females at diagnosis. However, these differences were more prominent in younger age groups [3]. FBG was significantly different between males and females below 45 years old and non-significantly different in those older than 45 years, with the mean being higher in males in all groups except those above 65 years old. According to Fangjian’s study, FBG strongly correlated with HbA1c and two-hour glucose, explaining why it is higher in males across all age groups. This correlation is significant in males below 45 years, who had a significantly higher HbA1c. However, it does not explain why this result is non-significant at ages above 45 years [22].

RBG had a higher mean in males for all groups, with a significant difference observed in the 35-44, 45-54, and above 65 age groups. The higher RBG in males than females is consistent with the findings of many previous studies that found RBG to be strongly and significantly correlated with HbA1c [23]. However, this does not explain why it is non-significant in those less than 35 years and above 55 years. The retrospective study design may limit the generalizability of the results since the sample population only included individuals who visited the center and had their lipid profiles measured. Furthermore, the retrospective design and the frequent missing of data about socioeconomic status, lifestyle habits, or comorbidities prevented the analysis of their impact on the study results. However, the study's large sample size increased its statistical power and enabled the investigation of subgroups of interest. Furthermore, the retrospective design's inability to establish causality hindered the determination of whether sex or other related factors were responsible for the observed differences in lipid profile findings between sexes. To address this, future studies may use a longitudinal study design to examine the temporal relationship between sex and lipid profile findings and include data from multiple centers to enhance the generalizability of the findings to diverse populations and settings.

## Conclusions

In newly diagnosed T2DM patients under 55 years old, males were significantly more likely to have higher HbA1c, FBG, RBG, TG, and lower HDL-C than females at the time of T2DM diagnosis. These differences disappeared in individuals aged 55 years and older, except for HDL-C, which continued to be lower. In females, the BMI at the diagnosis of T2DM was significantly higher than in males across all age groups except for those under 35 years old. These findings suggest that young males with T2DM are at a higher risk of cardiovascular complications compared to females in the same age group.
